# Nine-month Trend of Time-Varying Reproduction Numbers of COVID-19 in West of Iran

**DOI:** 10.34172/jrhs.2021.54

**Published:** 2021-06-28

**Authors:** Ebrahim Rahimi, Seyed Saeed Hashemi Nazari, Yaser Mokhayeri, Asaad Sharhani, Rasool Mohammadi

**Affiliations:** ^1^Department of Public Health, Mamasani Higher Education Complex for Health, Shiraz University of Medical Sciences, Shiraz, Iran; ^2^Prevention of Cardiovascular Disease Research Center, Department of Epidemiology, School of Public Health and Safety, Shahid Beheshti University of Medical Sciences, Tehran, Iran; ^3^Cardiovascular Research Center, Shahid Rahimi Hospital, Lorestan University of Medical Sciences, Khorramabad, Iran; ^4^Department of Epidemiology, School of Public Health, Ahvaz Jundishapur University of Medical Sciences, Ahvaz, Iran; ^5^Department of Biostatistics and Epidemiology, School of Public Health and Nutrition, Lorestan University of Medical Sciences, Khorramabad, Iran; ^6^Nutritional Health Research Center, Health and Nutritional Department, Lorestan University of Medical Sciences, Khorramabad, Iran

**Keywords:** Basic reproduction number, COVID-19, Transmissibility Measures, Disease Transmission, Infectious, Iran

## Abstract

**Background:** The basic reproduction number (R_0_) is an important concept in infectious disease epidemiology and the most important parameter to determine the transmissibility of a pathogen. This study aimed to estimate the nine-month trend of time-varying R of COVID-19 epidemic using the serial interval (SI) and Markov Chain Monte Carlo in Lorestan, west of Iran.

**Study design:** Descriptive study.

**Methods:** This study was conducted based on a cross-sectional method. The SI distribution was extracted from data and log-normal, Weibull, and Gamma models were fitted. The estimation of time-varying R_0_, a likelihood-based model was applied, which uses pairs of cases to estimate relative likelihood.

**Results:** In this study, R_t_ was estimated for SI 7-day and 14-day time-lapses from 27 February-14 November 2020. To check the robustness of the R_0_ estimations, sensitivity analysis was performed using different SI distributions to estimate the reproduction number in 7-day and 14-day time-lapses. The R_0_ ranged from 0.56 to 4.97 and 0.76 to 2.47 for 7-day and 14-day time-lapses. The doubling time was estimated to be 75.51 days (95% CI: 70.41, 81.41).

**Conclusions:** Low R_0_ of COVID-19 in some periods in Lorestan, west of Iran, could be an indication of preventive interventions, namely quarantine and isolation. To control the spread of the disease, the reproduction number should be reduced by decreasing the transmission and contact rates and shortening the infectious period.

## Introduction

In the last days leading up to the end of 2019, the Chinese government officially announced the outbreak of an epidemic with a new strain of the corona family, later called the coronavirus disease 2019 (COVID-19) ^1^. In the following weeks, infections spread across China and other countries around the world ^2^. Therefore, estimating epidemiological parameters of COVID-19, such as the basic reproduction number (R_0_), has a pivotal role in predicting its trend ^3^.

The R_0_ is an important concept in the epidemiology of infectious disease and the most important parameter to determine the transmissibility of a pathogen ^4^. By definition, R_0_ refers to the average number of susceptible individuals who develop the disease following contact with an infected individual over his or her infectious period. This index shows the power of infectious disease transmission ^5^. On the other hand, R_0_ is a theoretical parameter that provides information about the spread rate of infectious disease in various stages of the epidemic among the susceptible population ^6^. Periodic evaluation and comparison of R_0_ or R not only help understand the dynamics of transmission and evolution but also are crucial to design or modify public health intervention strategies. In infectious disease epidemiology, the average number of secondary cases that will occur in a mixed susceptible and non-susceptible host population when one infected individual is introduced. Its relationship with *R*_0_ is given by * R *= * R*_0_ x where *x *is the proportion of the host population that is susceptible ^7^. The goal of effective control measures is the suppression of the epidemic ^8^. The basic reproduction number can be influenced by a variety of factors, including the likelihood of transmission during contact between infected and susceptible people, the frequency of contact, and the duration of infection in the individual ^6^. The value of R_0_ varies over time during an epidemic of infectious disease. Therefore, this investigation was conducted to estimate the nine-month trend of time-varying R (Rt) of COVID-19 epidemic using the serial interval (SI) and Markov Chain Monte Carlo (MCMC) in Lorestan, west of Iran.

## Methods

 This study was conducted in Lorestan Province, west of Iran, and approved by the Institutional Review Board of Lorestan University of Medical Sciences, Lorestan, Iran (IR.LUMS.REC.1399.001). Two sources of data for this study were public health sectors and the special unit of the hospital, which admitted the cases. The Deputy of Health in the Ministry of Health and Medical Education in Iran runs a phone screening program by public health sectors. Additionally, the emergency unit of referral hospitals collected data for patients with serious conditions. Consequently, data for inpatient and outpatient cases were obtained. The researchers extracted daily infections and information about the primary and secondary cases. Moreover, moving average smoothing with a span of 5 was used for all cases. Calculating the time-varying R, all confirmed, probable, and suspected cases of COVID-19, including inpatient and outpatient cases, were entered into the study. A confirmed case was a patient whose real-time reverse transcription polymerase chain reaction (RT-PCR) was positive. A probable case had clinical criteria with close contact to a confirmed case of COVID-19 within 14 days from the onset of symptoms or had a positive lung computed tomography scan. The suspected case had merely clinical symptoms. Totally, 52,946 cases were registered in the Lorestan Province within 27 February-14 November 2020.

Calculating the Rt generation time (GT) was needed, however, when GT was inaccessible and unknown, SI (i.e. the interval between clinical onset in the initial case and secondary case) was used instead ^9,10^. The researchers extracted SI distribution from data and fitted log-normal, Weibull, and Gamma models using close contact of patients. The application of the “est.GT” function in R_0_ package led to the production of the best model. The Bayesian approach was used to estimate the time-varying R_0_ in the west of Iran. To estimation the time-varying R_0_, the method developed by Cori et al. (2013) was used ^8^. This likelihood-based model uses pairs of cases to estimate relative likelihood. The confidence interval (CI) was obtained by 5,000 simulations. The EpiEstim R package software (version 2.2-3) was employed to analyze data and estimate the time-varying R_0_. In the EpiEstim package, a Bayesian methodology was used to estimate the time-varying R_0_. This methodology applied the Metropolis algorithm to obtain MCMC.

Since the reproduction number is sensitive to GT, two scenarios were used to calculate the R_t_. the researchers estimated R_t_ from 27 February-14 November 2020 for 7-day and 14-day serial intervals. To check the robustness of the R_0_ estimations, sensitivity analysis was performed using different SI distributions to calculate reproduction number in 7-day and 14-day time-lapses.

## Results

**Figure 1:**
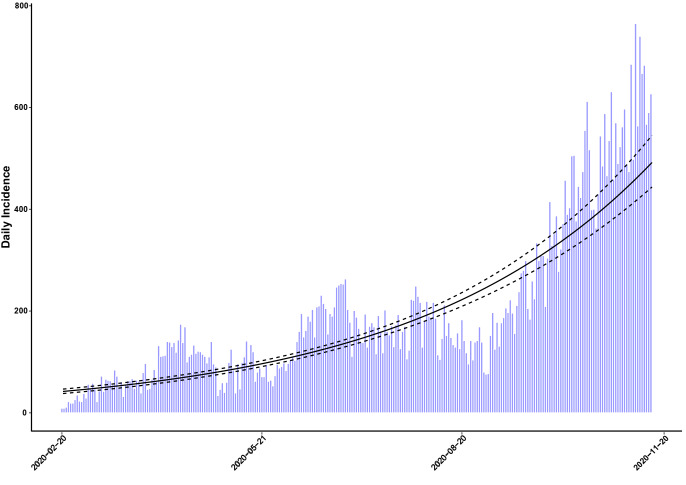
Epidemic curve of COVID-19 in Lorestan, Iran, within 22 February-14 November 2020

Within 27 February-14 November, 52,946 confirmed, probable, and suspected COVID-19 cases were recorded in Lorestan Province. The epidemic curve of daily cases showed a considerable peak around November (Figure 1). Doubling time (i.e., the time required for the number of cases to be doubled) during the study period was obtained at 75.51 days (95% CI: 70.41-81.41).

**Figure 2:**
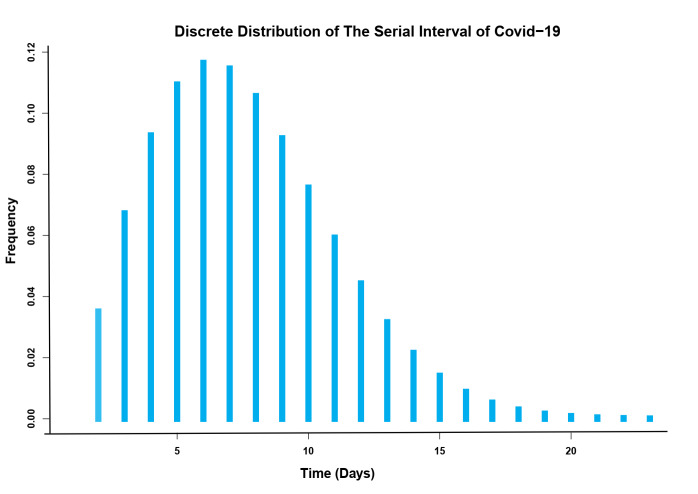
Distribution of serial interval of COVID-19 in Lorestan, Iran

To estimate SI, close contact of 14,262 confirmed COVID-19 cases by RT-PCR test were followed. Of these, 1,677 infector-infectee transmission pairs were identified. For each transmission pair, the time between the symptom onset dates of the infector and infectee was measured. Several distributions were examined on SI, and Weibull distribution was the best-fit model with a mean score of 6.55±3.40 days (Figure 2).

**Figure 3:**
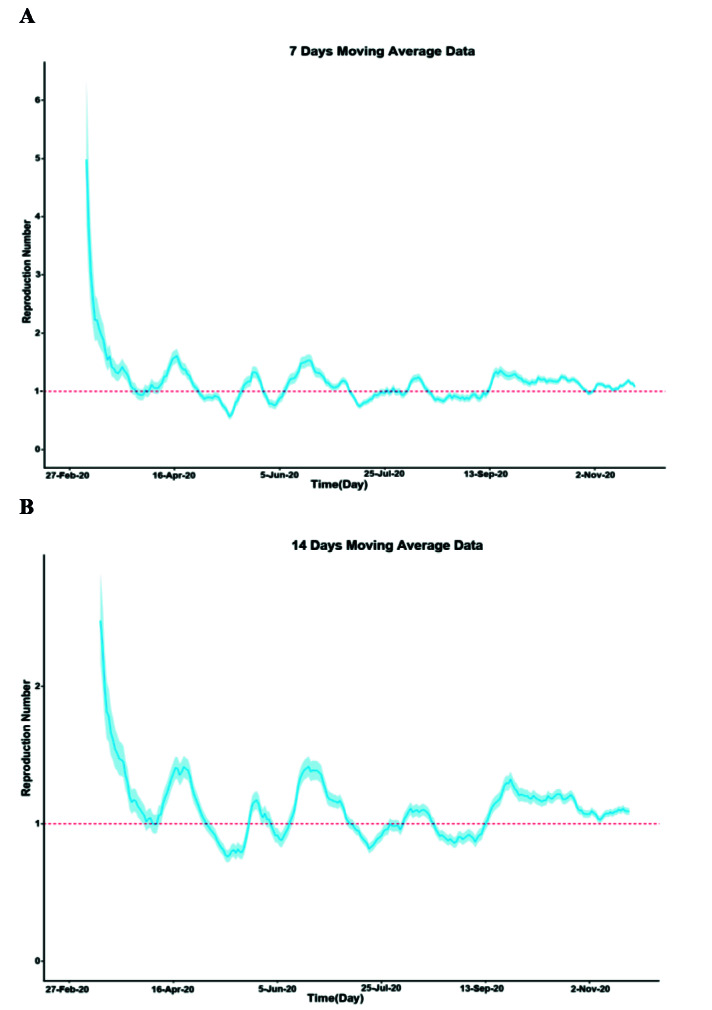
Time-varying reproduction number (95% confidence interval) of COVID 19 in Lorestan, Iran, using Markov Chain Monte Carlo models in time-lapses of 7 days (**A**) and 14 days (**B**)

**Figure 4:**
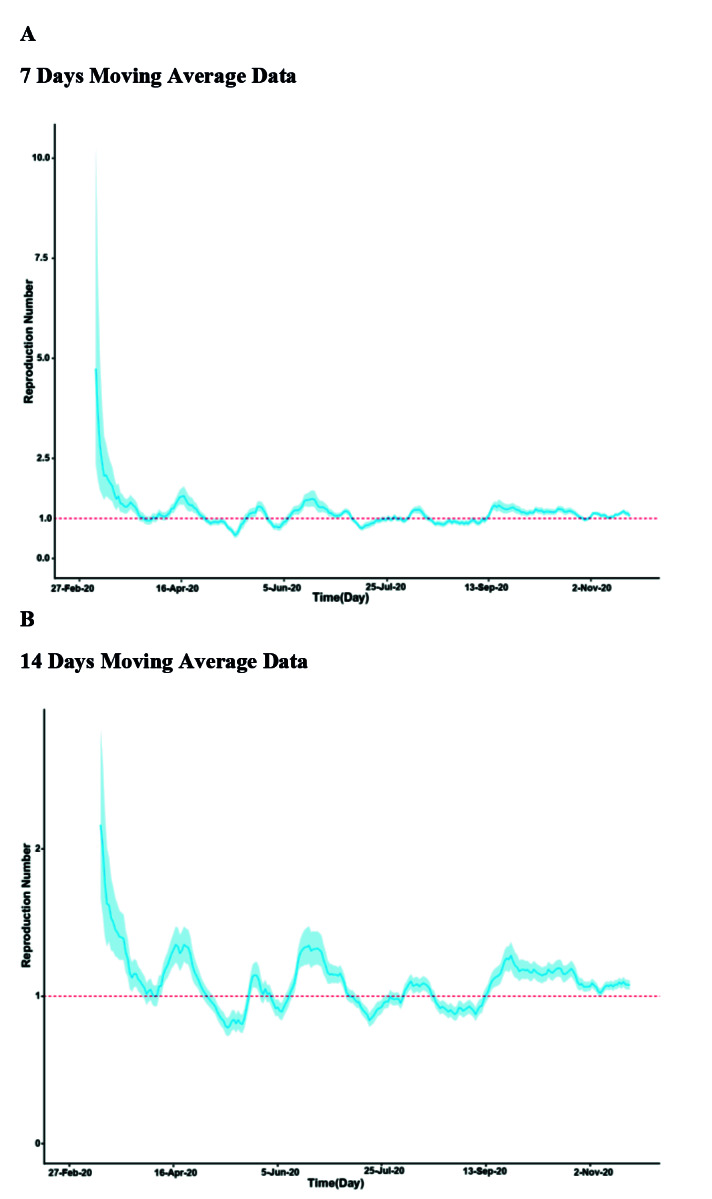
Sensitivity analysis for time-varying reproduction number (95% confidence interval) of COVID-19 in Lorestan, Iran, in time-lapses of 7 days (**A**) and 14 days (**B**)

Figure 3 displays estimated time-varying reproduction numbers (Rt) values with 95% CI for two-time lapses of 7 and 14 days, panels A and B, respectively. The R(t) ranged from 0.56 to 4.97 for 7-day time-lapse and from 0.76 to 2.47 for 14-day time-lapse. For both 7-day and 14-day time lapses, the minimum values were obtained in May. The R(t) dropped below one in value sometimes during the study period. However, its waves tended to be smoothed during the time and implied fairly lower transmissibility, compared to the early stage of the epidemic. A sensitivity analysis was conducted to determine the effect of SI changes on estimated R in two-time lapses of 7 and 14 days. For both 7-day and 14-day time lapses, estimated R values for COVID-19 were robust to the changes in SI parameters (Figure 4).

## Discussion

The present study aimed to estimate the SI and R_0 _of COVID-19 in Lorestan Province for a nine-month period. According to our findings, the mean SI was estimated at 6.55 days. This result is in line with those of most pieces of research suggesting the range mean SI for COVID 19 at 4-8 days ^11-13^. Based on the results of a systematic review and meta-analysis, a pooled estimate was 5.19 days ^14^. Another study estimated this value as 3.96 days, which was lower than that of the present study ^15^.

In Iran, a similar study estimated the mean of SI at 5.71 days ^16^. In another study conducted by Aghaali et al., this value was calculated at 4.55 days, in which different statistical methods were used to calculate the value in central Iran ^17^. An estimated SI was obtained at 3.96 days based on 468 reported transmission pairs ^15^, which was lower than the reported mean estimate in the present study.

Numerous factors, including the nature of contacts, rate of incidence, strategy of testing, and onset of control measures, may contribute to the observed differences ^18-21^. The adopted estimation methods of SI and the number of transmission pairs can also affect the results, which may be different in various studies. However, using real-time estimation of SIs provides more accurate estimation ^22^.

The number of R(t) in this study was estimated at the ranges of 0.56-4.97 and 0.76-2.47 for 7-day and 14-day time lapses. Consistent with this finding, most studies, including the 11^th^ report of the National Epidemiological Committee of COVID-19, have estimated a number between 2 and 5 ^12,17,23,26^. Nevertheless, to the best of our knowledge, few studies have reported this value higher than 5 ^27,28^. Additionally, in the line with the 11^th^ report of the National Epidemiological Committee of COVID-19 ^26^, our findings showed that following an initial high value, the R(t) dropped below one sometimes during the study period. It indicated that the control measure carried out both by the provincial government and community members was effective. A package of control measures, including isolating cases, quarantining contacts, restricting borders, changing population behavior (e.g., physical distancing), and adopting personal protective measures, that Iran has implemented at the early stage of the pandemic, is associated with the reduced spread of COVID-19. These measures were also followed in Lorestan Province, where the present study was conducted.

At the beginning of April 2020, the local government decided to lift some restrictions gradually. Some businesses were allowed to resume operations cautiously. However, this measure reduced the sensitivity of individuals and families to the issue, which can be considered a possible reason for the epidemic curve with a peak around mid-April 2020. It seems that an important factor that led people to follow the health protocols in early April was the realization of the seriousness of the health issue ahead. One of the main challenges in the COVID-19 epidemic has been effective and timely intersectoral coordination. To translate COVID-19 data into information and consequently into policy, it is necessary to establish extensive intersectoral collaboration ^29^.

The COVID-19 crisis and its consequences have highlighted the importance of behavior changes ^30^. As mentioned in a study conducted by Bavel et al. ^31^, the reduction of viral transmission during epidemics requires considerable changes in behavior. The COVID-19 is not exempt. In the absence of effective treatment and vaccine, wearing a face mask, washing hands, staying at home, and observing physical distance, along with government containment measures, will play an effective role in preventing the disease at the individual level and reducing its trend at the society level. People will be challenged to do these behaviors ^32^. To change behaviors in this area, it is necessary to consider the novel coronavirus as a threat and realize that the acceptance of new behaviors will have benefits and positive consequences. In this way, social media plays an important role in convincing people in this regard.

The present study had some limitations. It was assumed that all daily cases of COVID-19 in our data were detected. In the study, asymptomatic or mild cases might have not been identified, especially in the early stage of the epidemic. This might have led to reporting patients with severe disease, and therefore, underestimate the actual number of cases. Accordingly, the findings of the present study should be interpreted with caution, which requires further validation. The swift isolation of such severe cases might have led to shorter SIs, potentially shifting those downward ^15^. Furthermore, given that the data about the timing of symptom onset were collected retrospectively, there was a possibility of recall bias; consequently, the cases might be for recent encounters rather than past encounters. Regarding this, the estimated SIs might be biased downward as well. However, the distribution of SIs could be varied across the epidemic ^15,33^.

## Conclusion

 Low R(t) of COVID-19 in some period time in Lorestan Province could be an indication of preventive interventions, namely quarantine and isolation. To control the spread of the disease, the reproduction number should be reduced by decreasing the transmission and contact rates and shortening the infectious period. Therefore, further attention on preventive approaches is highly recommended.

## Acknowledgments

 The authors are grateful to all those researchers who contributed to conducting this study. This work was approved by the Institutional Review Board at the Lorestan University of Medical Sciences and supported by research grants 1399001.

## Conflict of interests

 The authors declare that there is no conflict of interest.

## Funding

 This study was financially supported by the Lorestan University of Medical Sciences with grant number 1399001.

## Highlights

- Several distributions were examined on the serial interval, and Weibull distribution was the best-fit model with a mean of 6.55±3.40 days.
- The number of Rt in this study was estimated at the ranges of 0.56-4.97 and 0.76-2.47 for 7- day and 14-day time lapses, respectively.
- Doubling time (i.e., the time required for the number of cases to be doubled) was obtained at 75.51 days.
- Following an initial high value, the R(t) dropped below one sometimes during the study period.
